# The Two Doctors, or the Old and New School

**Published:** 1840-07

**Authors:** 


### THE TWO DOCTORS, OR THE OLD AND NEW SCHOOL.

"Look here upon this picture, and on this:
The counterfeit presentment of two brothers."

Et ces pauvres médecins! que n'a-t-on pas dit de leur air doctoral et de leurs manières empesées! Molière ne s'amusait que de leur ignorance: mais le monde, qui, cependant, croyait en eux, se moquait de leur gravité. On les accusait de faire étalage de leur savoir, de n'employer que des mots baroques, et de nous envoyer dans l'autre monde avec des adieux inintelligibles. Comme on a ride leur lourde perruque et de leur canne à pomme d'or! Que de fois on a dit: le pédant docteur! l'ennuyeux docteur! le lourd disciple d'Hippocrate! Ces épigrammies n'auraient plus de sens aujourd'hui que nos plus amusans causeurs sont nos médecins; comment ne pas se divertir de leurs piquantes anecdotes, còntées avec tant d'esprit? mais aussi comment prendre au sérieux les ordonnances d'un médecin si amusant? On oublie de lui expliquer ses souffrances en l'écoutant. Oh! les médecins ne sont plus d'ennuyeux docteurs; aujourd'hui, ils sont, au contraire, très-aimables; hélas! trop aimables; et, en cela, ils sont plus cruels que leurs prédécesseurs, car, s'ils vous laissent mourir comme eux, ils vous font bien amèrement regretter une existence que leur intéressant entretien vous rendait si agréable.

Vicomte Charles De Launay.—*Le Courrier de l'Europe*, 6 *Juin*, 1840.*

* A most excellent French newspaper, published in London.

